# Highly Porous Organic Polymers for Hydrogen Fuel Storage

**DOI:** 10.3390/polym11040690

**Published:** 2019-04-16

**Authors:** Kimberley Cousins, Renwu Zhang

**Affiliations:** Department of Chemistry and Biochemistry, California State University, San Bernardino, CA 5500, USA; KCousins@csusb.edu

**Keywords:** H_2_ storage, porous organic polymers, hypercrosslinked polymers (HCPs), polymers of intrinsic microporosity (PIMs), conjugated microporous polymers (CMPs), porous aromatic frameworks (PAFs)

## Abstract

Hydrogen (H_2_) is one of the best candidates to replace current petroleum energy resources due to its rich abundance and clean combustion. However, the storage of H_2_ presents a major challenge. There are two methods for storing H_2_ fuel, chemical and physical, both of which have some advantages and disadvantages. In physical storage, highly porous organic polymers are of particular interest, since they are low cost, easy to scale up, metal-free, and environmentally friendly. In this review, highly porous polymers for H_2_ fuel storage are examined from five perspectives: (a) brief comparison of H_2_ storage in highly porous polymers and other storage media; (b) theoretical considerations of the physical storage of H_2_ molecules in porous polymers; (c) H_2_ storage in different classes of highly porous organic polymers; (d) characterization of microporosity in these polymers; and (e) future developments for highly porous organic polymers for H_2_ fuel storage. These topics will provide an introductory overview of highly porous organic polymers in H_2_ fuel storage.

## 1. Introduction

The world is reaching its “Oil Peak” and petroleum resources are expected to be exhausted in coming decades. Seeking alternatives to fossil fuels is imperative. H_2_ is thought to be a viable alternative fuel due to its large abundance and clean combustion. In addition, H_2_ has a much higher energy density (142 kJ/g) than that of petroleum oil (47 kJ/g) [1]. However, the use of H_2_ as a fuel in automobiles has a major obstacle: the onboard storage of H_2_ gas. Many methods to store hydrogen have been proposed. The most direct way is to storage H_2_ as a liquid or a high-pressure gas [2,3]. This approach requires significant energy for liquefying or pressurizing H_2_ gas, which has a very low boiling temperature (20 K) and critical temperature (33 K), and also poses many safety concerns due to extremely low temperature and high pressure. In addition, H_2_ can embrittle steel gas tanks after long storage, generating additional risk. As an alternative, H_2_ storage media have been extensively explored, under the H_2_ program initiated by the U.S. Department of Energy (DOE) in 2003. The DOE set several criteria for materials for onboard H_2_ storage: (1) high storage capability, that is, 5.5 wt % and 40 g/L at ambient conditions; (2) rapid H_2_ release and recharge under moderate conditions; and (3) long recycling life, that is, more than 1000 recharge and discharge cycles [4,5]. The first two criteria have proved particular challenging, so that the DOE has postponed its target date several times. For instance, the DOE set its initial target for 2015, extended it to 2017, and then to 2020 [6]. The ultimate goal of the DOE is 6.5 wt % and 60 g/L by 2050, as summarized in Table 1 [7].

As seen from Table 1, there are many engineering and economic requirements, as well as scientific requirements for hydrogen fuel to be successfully employed as an onboard energy power for vehicles. In order to be adopted commercially, a hydrogen storage medium must meet all above criteria. Nevertheless, two fundamental scientific criteria, gravimetric capacity, and volumetric capacity, are the primary and most important parameters for scientists to pursue. Therefore, in this review, we will focus on progress made in meeting the first two criteria, particularly the gravimetric capacity, using porous organic polymers for the hydrogen storage.

Currently there are two major ways to store H_2_ molecules: (a) chemical absorption by forming hydrogen-containing molecules and (b) physical adsorption in highly porous materials. The former approach includes metal hydrides such as NaH, LiH, NaAlH_4_ [8,9,10], etc. as well as other hydrogen-containing molecules such as H_3_N–BH_3_ [11]. These materials store H_2_ via chemical bonding. The advantage of these materials is that they have a relatively high storage capability. The disadvantage is that they require high temperatures to break the chemical bonds and release H_2_ molecules, so that the recovery of H_2_ gas from these chemicals is not energy efficient. Organic hydrogen carriers or organic hydrides are quite reactive, leading to safety concerns, and disposal of metals poses the environmental concerns. In contrast, physical adsorption uses weak van der Waals interactions, and therefore H_2_ molecules can be released easily under moderate conditions. However, due to these weak interactions, physical adsorption, which can retain relatively large amount of H_2_ at liquid nitrogen temperature, has greatly reduced storage capability at room temperature. Physical adsorption methods can be achieved with materials with high porosity, including metal organic frameworks (MOFs), covalent organic frameworks (COFs), activated carbons, carbon nanotubes, carbides, and highly porous polymers [12,13,14]. Currently, the general approach for using physical adsorption in H_2_ storage is to increase the internal surface area in materials. For instance, activated carbons can have surface areas of up to 2000 m^2^/g and store H_2_ up to 5 wt % at 77 K/40 bar [15], but storage capacity decreases to 1.0 wt % at 298 K/200 bar [16]. Single walled and multiwalled nanotubes can store 6 wt % at 77 K [17]. MOFs, which are crystalline solids consisting of multidentate organic ligands connecting to a metal ion, afford a very large internal surface area, e.g., 5000 m^2^/g [18]. A wide range of MOF materials have been synthesized. 

Porous organic polymers have great advantages over than other materials; for instance, highly porous polymers are more stable at the ambient conditions under which the metal ions in MOFs are sensitive the moisture. The polymeric structures can be well controlled via different organic synthetic routes and starting materials. Four groups of highly porous polymers have been studied intensively for hydrogen storage: (a) hypercrosslinked polymers (HCPs), (b) polymers of intrinsic microporosity (PIMs), (c) conjugated microporous polymers (CMPs), and (d) porous aromatic frameworks (PAFs). There are many excellent studies in this field that are making gradual progress towards the DOE hydrogen storage targets. Broom et al. provide an overview on porous materials for hydrogen storage, including practical considerations of the technology [19]. This review is not a summary of all of these works, rather, it starts from basic theory for H_2_ storage, and an overview of each storage medium, highlighting the most effective results, and then addresses the challenges and directions for developing highly porous polymers needed to meet DOE standards. The paper is arranged in the following structure: first, basic theoretical considerations for storing the maximum amount of H_2_ at ambient conditions are introduced; then the different types of highly porous polymers are summarized in terms their structure, surface area, porosity characterization, and hydrogen storage ability. Finally, future directions for investigation and possible effective solutions are proposed.

## 2. Theoretical Considerations

Theoretical background is introduced from two perspectives: first, we present the fundamental thermodynamic requirements for adsorption enthalpy needed to store the maximum amount of hydrogen, as well as releasing/adsorbing H_2_ molecules readily at ambient conditions as required by the DOE criteria; and second, we outline the enhancement of interaction between H_2_ molecules and the host cavity, using molecular energy level considerations, necessary to achieve the overall adsorption enthalpy required.

Probably the most promising theoretical work on the physisorption of H_2_ from the thermodynamic perspective was carried out by Bhatia et al., who were motivated by the conflicting reports on the capacity of H_2_ storage in highly porous materials [20]. For instance, an isoreticular metal organic framework (IRMOF) material, IRMOF-8, was first reported to have an H_2_ absorption capacity of 2.0 wt % H_2_ at 298 K/10 bar [21]. However, subsequent computer simulations from Rowsell et al. predicted a H_2_ uptake of only 0.75 wt % at 77 K/1 bar for IRMOF-8, suggesting a significantly lower capacity [22]. In order to explore the theoretical limit for the maximum delivery of H_2_, which is the difference between H_2_ in a charged and discharged material, Bhatia et al. used the Langmuir model to model H_2_ adsorption inside macropores/mesopores (pores with diameters of 2–50 nm and of >50 nm, respectively) of materials to find the amount of H_2_ adsorbed under equilibrium conditions, with an equilibrium constant *K* as shown in Equation (1).

(1) $$n=\frac{KPn_{m}}{1+KP},$$

where *K* is equilibrium constant of the adsorption, *P* is the pressure of H_2_ gases, *n* is the amount of H_2_ adsorbed and *n_m_* is the quantity of active adsorbing sites in a material.

So the delivery (*D*) of H_2_ under two different pressures, *P*_1_ and *P*_2_, is:

(2) $$D(K,P_{1},P_{2})=n_{1}-n_{2}=\frac{KP_{1}n_{m}}{1+KP_{1}}-\frac{KP_{2}n_{m}}{1+KP_{2}}.$$

When *P*_1_ and *P*_2_ are fixed, the equilibrium constant *K* for the maximum delivery (*D*) can be found by setting *dD*/*dK* = 0 in Equation (2) to give Equation (3):

(3) $$K=\frac{1}{\sqrt{P_{1}P_{2}}}.$$

According to the Gibbs equation:

*lnK* = −Δ*G*°/*RT* = − (Δ*H*°− *T*Δ*S*°)/*R*T. (4)

By combining Equations (3) and (4), the optimal adsorption $\Delta H_{ad}^{\circ }$ for the maximum delivery (*D*) is obtained as Equation (5):

(5) $$\Delta H_{opt}^{\circ }=T\Delta S_{}^{\circ }=\frac{RT}{2}\ln\left(\frac{P_{1}P_{2}}{P_{0}^{2}}\right).$$

Equation (5) can be used to search for the optimal $\Delta H_{ad}^{\circ }$ for different adsorption materials. On the other hand, for the same material, the optimal adsorption temperature $\Delta T_{opt}^{}$ can be determined by rearranging Equation (5) to give Equation (6):

(6) $$T_{opt}^{}=\frac{\Delta H_{}^{\circ }}{[\Delta S_{}^{\circ }+(R/2)\ln(P_{1}P_{2}/P_{o}^{2})]},$$

where ΔS° ≈ −8*R* for H_2_ adsorption in Langmuir model. If H_2_ is charged/discharged at the pressures of 30/1.5 bar and the temperature of 77 K, $\Delta H_{opt}^{\circ }$ is calculated to be –6.3 kJ/mol, close to –5.0 kJ/mol reported recently for a variety of porous materials at cryogenic temperature [23]. The difference between these values might come from the recharge *P_1_* and discharge pressure *P_2_*. However, when storing H_2_ under the same pressures, but at 298 K as DOE expects, the calculated $\Delta H_{opt}^{\circ }$ is –15.1 kJ/mol. This value for the adsorption is optimal with respect to the affinity of hydrogen—strong enough to store a large amount of hydrogen gas at the charging pressure (~30 bar) but weak enough to release most of that hydrogen at the discharge pressure (~1.5 bar). Unfortunately, for most physical adsorbents, the heat of adsorption is much less that this value. For instance, for activated carbons or hydrocarbons, the adsorption enthalpy is only about −5.6 kJ/mol [24], so that adsorption of hydrogen on carbon-related materials is too weak for storing large amount of hydrogen at ambient temperature. Similar values and conclusions have also been applied to other porous materials such as zeolites [25] and metal organic frameworks (MOFs) [26]. 

On the other hand, if $\Delta H^{\circ }$ = −5.6 kJ/mol for hydrocarbons, including polymers, is used in Equation (6), the calculated optimal temperature for highest delivery *T_opt_* = 114.4 K, which is much lower than the ambient temperature needed to meet the DOE specifications. This analysis explains why most highly porous materials, including polymers, can adsorb a relatively large amount of H_2_ at liquid nitrogen temperature (77K), some of them up to 8.0 wt %, but the capacity drops to <1.0 wt % under ambient conditions.

**Figure 1:**
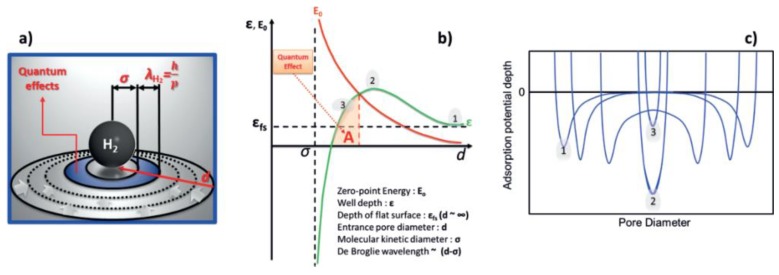
Theoretical simulation of adsorption potential for H_2_ ultra micropores with a few Å in radius: (**a**) schematic diagram of quantum sieving effect, (**b**) behavior of interaction potential, (**c**) adsorption potential versus pore size (Reproduced with permission from Ref. [32]. Copyright @ 2016 John Wiley and Sons).

Therefore, to increase H_2_ adsorption ability at room temperature via physisorption, we must increase the adsorption enthalpy $\Delta H^{\circ }$. To achieve this, several methods from the theoretical perspective have been proposed. A first approach is to create as many ultra micropores (<1 nm) at the atomic scale as possible. Theoretical simulations show that at low pressure and high temperature (desired conditions of the DOE), graphite with an inter-layer distance of 6 Å (0.6 nm) has the highest adsorption capability, of above 10 kJ/mol, achieved through the overlap of adsorption potentials from the opposite walls [27]. In addition to such 1D slit pores, 2D cylindrical, and 3D spherical pores exhibit the same principle: cylindrical pores with *r* ≈ 2.0 Å and spherical pores with *r* ≈ 3.8 Å have maximum adsorption abilities [28,29]. Furthermore, although the free H_2_ molecules at room temperature behavior classically, when localized in ultra micropores, H_2_ molecules manifest quantum confinement behavior via the quantum sieving effect [30,31], which persists up to 300 K [26]. The quantum sieving effect versus the pore size is illustrated in Figure 1, in which the optimal pore diameter, on the order of the de Broglie wavelength for H_2_, corresponds to the point in which both sides of the pore interact with the absorbed molecule (corresponding to Figure 1a, blue band λ_H2_; Figure 1b, region A; and point 2 on Figure 1c) [32].

**Figure 2:**
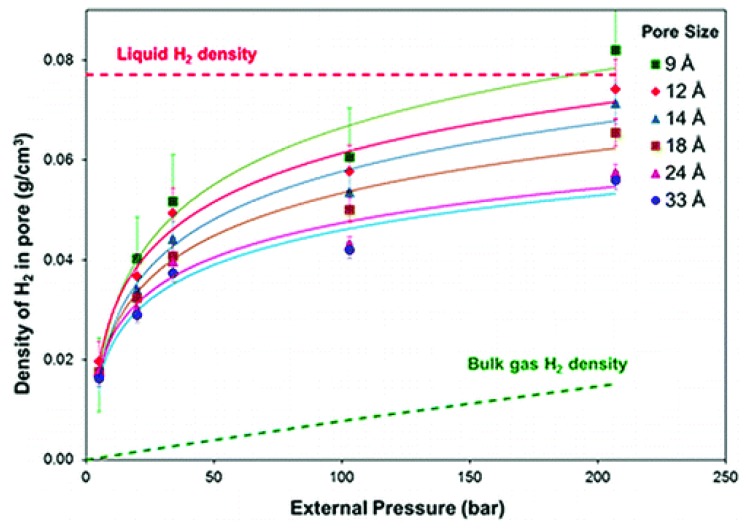
H_2_ adsorption in pores with different sizes studied via in-situ neutron scattering (Reproduced with permission from [33]. Copyright @ 2011 ACS publications).

The theoretical predictions for enhancement of H_2_ adsorption due to ultra micropores have been demonstrated by the experiment. For example, Gallego et al., employed in-situ small-angle neutron scattering to study H_2_ adsorption in activated carbons with different pore sizes, and concluded that the smaller the pore size, the larger the absorbed H_2_ density, as shown in Figure 2 [33]. More recently, Lee and coworkers reported that hard carbon materials with the largest micropore (<1.05 nm) volume showed the greatest H_2_ uptake at ambient temperature and high pressure [34]. It is of particular interest to find from the figure that the density of adsorbed H_2_ inside pores with 9 Å is almost same as the liquid H_2_ density at 298 K and 200 bar.

**Figure 3:**
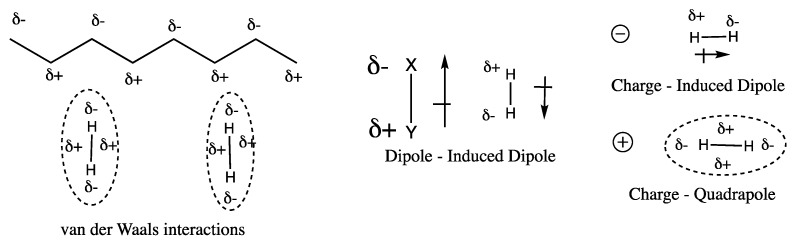
Schematic diagram of the different types of interactions between an H_2_ molecule and absorbent materials.

A second approach to improve adsorption enthalpy is to introduce charge sites in porous materials, creating charge-induced interaction between H_2_ and adsorbent [35]. At the molecular level, H_2_ molecules interact with each other or with other non-polar molecules such as hydrocarbon polymers via quadropole–quadropole interaction due to the London dispersion effect and generate a very weak van der Waals attractive force. However, when charged sites are present, additional charge-induced forces, such as dipole-induced dipole interactions, are generated, as shown in the schematic diagram Figure 3. The dependence of interactions with the distance of H_2_ from a host site, as well as the magnitude of interactions is shown in Table 2. The sum of the interactions determines the overall adsorption enthalpy $\Delta H^{\circ }$ of H_2_ molecules. 

A third approach to increase absorption enthalpy is to create orbital interactions between an H_2_ molecule and *d*-orbitals of a transition metal intercalated in the highly porous materials [36]. The typical adsorption enthalpy from orbital interactions is 20–160 kJ/mol and therefore exceeds the optimal value for the maximum delivery of H_2_. This adsorption of H_2_ is on the order of a chemical adsorption mechanism. Such metals are typically intercalated in covalent organic frameworks (COFs), which are technically classified as molecular crystals rather than polymeric materials, and hence are beyond the scope of this review.

Although the adsorption enthalpy is the key parameter needed to retain H_2_ molecules at room temperature, the internal surface area of micropores is another essential parameter in storing needed quantities of H_2_. Internal surface area is paramount, as the critical temperature of H_2_ (22 K) is well below the most practical cryogenic temperature afforded by liquid nitrogen temperature (77 K). Consequently, even at liquid nitrogen temperature, only a single layer of H_2_ molecules is adsorbed on the internal surface of micropores in highly porous materials, including polymers. This is why the Langmuir model, which is based on the single layer adsorption, can be used to obtain the optimal adsorption enthalpy $\Delta H^{\circ }$. The presumption of the Langmuir model is that adsorption ability is proportional to the number of active adsorbing sites, which are in turn proportional to the total surface area of the internal wall of micropores. Therefore, most published reports for H_2_ storage polymers optimize high internal surface area through synthetic strategies and/or physical methods. The corresponding H_2_ adsorption ability is measured at liquid nitrogen temperature (77 K), not ambient temperatures needed for practical use. In the following section, we review different classes of highly porous polymers with large internal surface areas, achieved by employing different synthetic methods and by adding different functional groups.

## 3. Highly Porous Organic Polymers for H_2_ Storage

**Figure 4:**
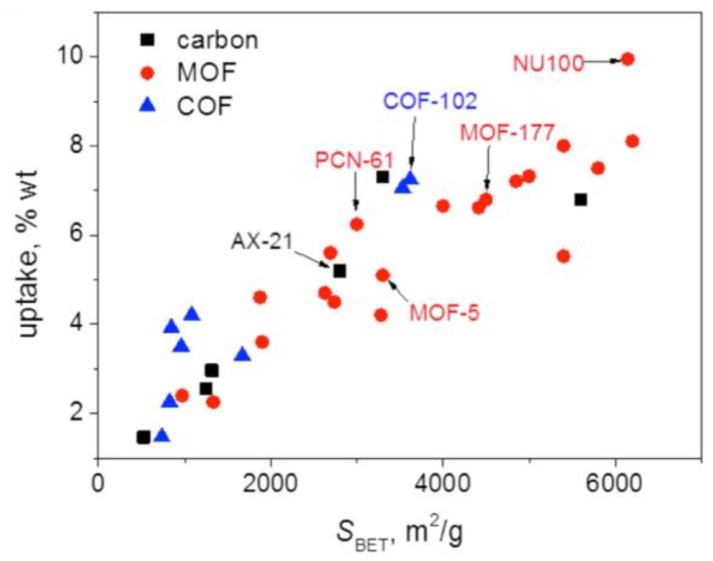
Comparison of metal organic frameworks (MOFs), covalent organic frameworks (COFs), and active carbons in H_2_ storage at 77 K (Reproduced with permission from Ref. [50]. Copyright @ 2016 Elsevier).

Several types of highly porous materials for H_2_ storage, other than organic polymers have been explored, with the aim of meeting the DOE criteria. They include: (a) Activated carbon and its modifications: a variety of methods were used to generate different types of activated carbon with large internal surface areas [37], as well as doping the carbons with different elements such as Pt, Pd, Rh, Ni, and Cu to enhance the adsorption enthalpy [38]. Surface area of some specially-activated carbons, e.g. open carbon frameworks (OCFs), can reach as high as 3800–6500 m^2^/g and excess H_2_ adsorption ability can reach 8.5 wt % at 77 K and 100 bar [39]; (b) Carbon nanotubes (CNTs): many studies have been performed on H_2_ storage in single walled (SWCNTs) or multiwalled carbon nanotubes (MWCNTs). The H_2_ storage capacity in CNTs, particularly SWCNTs with some metal doping, is relatively high, around 7.0–10 wt %, at 77 K. Some reports claim 14–20 wt % of storage at the temperature of 298 K or above, but these results have not been widely replicated [40]. There are some excellent reviews on the H_2_ storage in CNT materials [17,41]; (c) Metal organic frameworks (MOFs): MOFs are crystalline solids composed of multidentate organic ligands connecting to metal ions. The surface area of MOFs can be extremely high, e.g., 6200 m^2^/g [18,42]. A wide range of MOF materials have been synthesized [43,44]. Here are just a few examples: For instance, Zn_4_O(CO_2_)_6_ has an adsorption of 1.0 wt % at 298 K and 20 bar and 4.5–7.5% at 78 K and 0.8 bar; MOF-5 with composition Zn_4_O(BDC)_3_ (BDC = 1,4-benzenedicarboxylate) gives 4.5 wt % at 77 K and 1.0 wt % at 298 K and 20 bar [21]. MOFs have weak interactions and readily release H_2_, but storage capacity is low under ambient conditions; and (d) Covalent organic frameworks (COFs): COFs are similar to MOFs, but with organic covalent bonds linking the frameworks together. The internal surface area of COFs is generally smaller than that of MOFs, however, lower mass organic elements and better stability of the networks make COFs an attractive material for H_2_ storage [34,45]. Similar to all other porous carbon materials, while COFs are good candidates for storing H_2_ gas at low temperature, their performance at ambient temperatures is low. Recently, several theoretical simulations suggest that the intercalation of other elements in COFs can enhance the H_2_ adsorption ability [46,47,48,49]. Comparison of H_2_ storage in the above porous materials has been reviewed by Liu et al., and a summary plot is shown in Figure 4 [50]. As seen in Liu’s review, and illustrated in Figure 4, MOFs outperform COFs and activated carbon at 77 K due to their high surface area. However, when at room temperature, none of them can meet the DOE criteria due to their small adsorption enthalpy. In addition, these higher storage capacities are achieved at high pressure, not 1 bar as would be needed for practical applications. A compilation of data collected at 1 bar/77K demonstrates reduced H_2_ storage capacity for MOFs of between 1.87-2.25 wt % [51].

All the above-mentioned materials are highly porous organic or organometallic crystals. In the remainder of this review, we will focus on amorphous, porous organic polymers. The structures of these polymers are varied, and can be tuned by varying synthetic routes and monomers. The different types of porous organic polymers and their performances are summarized as follows.

### 3.1. Hypercrosslinked Polymers (HCPs)

**Figure 5:**
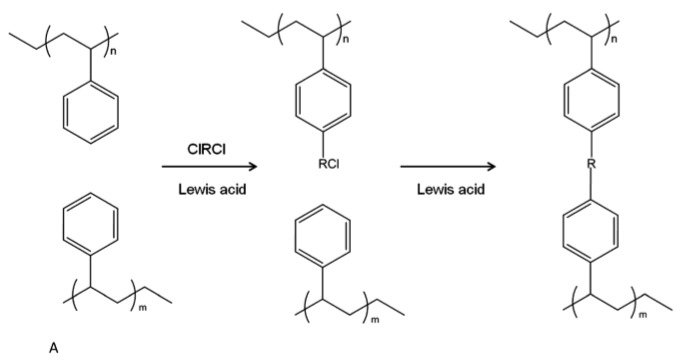
Basic synthetic route of hyper crosslinked polymers: polymers with aromatic side chains are linked using a high concentration of methylene dihalides in the presence of a Lewis acid catalyst, to create a high concentration of cross-links.

Hypercrosslinked polymers are co-polymers synthesized via the Friedel–Crafts method, e.g., poly(styrene-co-vinylbenzyl chloride) (PS-VBC) [52]. By choosing the right monomers, the polymer will retain networks with a very fine pore structure, and large internal surface area [53]. The surface area of this type of polymer can reach 2000 m^2^/g with pore sizes of 2–4 nm, and hydrogen storage capacity of 5 wt % at 77 K/80 bar, but only 0.2 wt % at 298 K/90 bar [54]. In addition to the high internal surface area, hypercrosslinked polymers also retain a high content of micropores. Large internal surface area and high micropore content make this type of polymer an attractive candidate for H_2_ storage. The basic synthetic route for hyper-cross linked polymers is shown in Figure 5. The benzene groups in polystyrene (PS) polymers are crosslinked by the crosslinkers, such as RCICClR’, and the microporosity within depends on the precursors used in the synthesis. When the initial precursor in making hypercrosslinked polymers is linear polystyrene (PS), as shown in Figure 5, the surface area can reach 1000 m^2^/g [55,56]. 

In addition to PS, other precursors have been used to create higher internal surface area; for instance, when Poly(vinylbenzyl chloride)-co-DVB (VBC-DVB) was used as the precursor, the internal surface area was extended to 1900 cm^2^/g, and the corresponding H_2_ storage ability at 77 K/15bar could reach 3.0 wt % [53]. Other precursors, such as polyanilines [57], polypyrroles [58], bischloromethyl monomers [59], etc. were also explored. The surface area for hypercrosslinked polymers based on these precursors can reach 632 m^2^/g, and the H_2_ storage ability at 77 K/30 bar reached 2.2 wt %. These values are significantly lower that the best MOFs and COFs a 77 K and high pressure, both of which possess much larger internal surface area. This is understandable since at 77 K, the surface area is the key parameter needed to store large amounts of H_2_ molecules. However, as the temperature increases to room temperature, both internal surface area and adsorption enthalpy play vital roles in storing and delivering the large amounts of H_2_ needed to meet DOE specifications, and hypercrosslinked polymers with more micropores could be competitive. In addition, at 1 bar pressure better-performing HPC’s have comparable H_2_ storage capacities to MOFs (see Table 3). Some typical hypercrosslinked polymers (HCPs) and their specific surface areas (SSAs) as well as H_2_ adsorption capacities are listed in Table 3. 

To enhance the gas adsorption, some metals, or metal complexes such as ferrocene, have been incorporated into hypercrosslinked polymers [60,61]. However, H_2_ storage ability did not improve significantly for these systems at 77K, remaining at about 1.0 wt %, probably due to the continued small internal surface area of about 1000 m^2^/g. In addition, other elements, such as Si, have also been included in the polymer structures to improve the thermal stability. However, there are only small changes in the surface area (~1200 m^2^/g) and H_2_ storage capacity (~1.25 wt %) at 77 K/1.12 bar [62,63]. The intercalated sulfur atoms in hypercrosslinked pitch samples provide incrementally better performance, with internal surfaces areas of 1377 m^2^/g, yielding 1.83 wt % H_2_ storage at 77 K/1.13 bar [64].

Most hypercrosslinked polymers are synthesized using metal catalysts. In contrast, hydroxy-group-containing porous organic polymers have been synthesized using organic catalysts. However, both surface area (up to 920 m2/g) and H_2_ adsorption capacity (up to 1.28 wt % at 77 K/1 bar) remain similar to other hydrocarbon hypercrosslinked polymers [73]. 

While most hypercrosslinked polymers were prepared using aromatic precursors, recently other hypercrosslinked polymers were derived from chlorinated polypropylene (CPP) grafted with polyethylenimine (PEI) via a hydrothermal amination reaction [74]. Different types of CPP-g-PEI co-polymers were synthesized. The H_2_ adsorption capacity was reported as high as 11.26 wt % at 77 K/50 bar and 2.47 wt % at 300 K/50 bar measured with commercial H_2_ storage analyzer (FineSorb-3110,) at 77K and 300 K. The H_2_ adsorption enthalpy of these CPP-g-PEI copolymers was calculated to be 38.79 kJ/mol, indicating that chemical, rather than physical adsorption may be in play. Thus, there might be a significant barrier to releasing H_2_ gas under ambient conditions. In contrast, the hypercrosslinked polymers that undergo physioadsorption show much lower H_2_ uptake at high pressure (3 wt % or less) on HCPs than on the best MOFs and COFs (up to 10 wt % at high pressure, as indicated in Section 2).

### 3.2. Polymers of Intrinsic Microporosity (PIMs)

**Figure 6:**
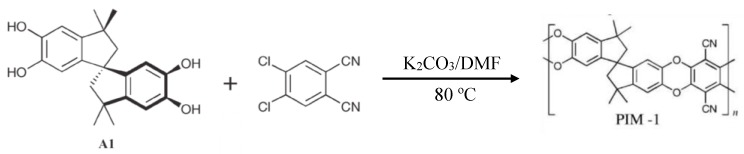
Basic synthetic route of polymers of intrinsic microporosity.

McKeown et al. initially developed PIMs by crosslinking close planar phthalocyanine macrocycles, which retain high surface area and contain rigid and nonlinear linkers preventing stacking of the monomers. The original crosslinker was derived from an agent, 5,59,6,69-tetrahydroxy-3,3,39,39-tetramethyl-1,19-spirobisindane, to make PIM-1 as shown in Figure 6 [75]. This coupling takes place under exceptionally mild reaction conditions, and in the absence of a transition metal. Other types of monomers and crosslinkers were also tried. However, it was found the PIM-1 retained the highest internal surface area, up to 1000 m^2^/g, and H_2_ adsorption ability was close to 1.7 wt % at 77 K and 10 bar. The high performance of PIM-1 has led to additional studies including aging and high pressures. For example, in a recent study Rochat et al. demonstrated that PIM-1 is stable over 400 days, showing only modest loss of hydrogen storage capacity, from 2.60 wt % (77 K/100 bar) on day 1 to 1.90 wt % on day 400 [76].

**Figure 7:**
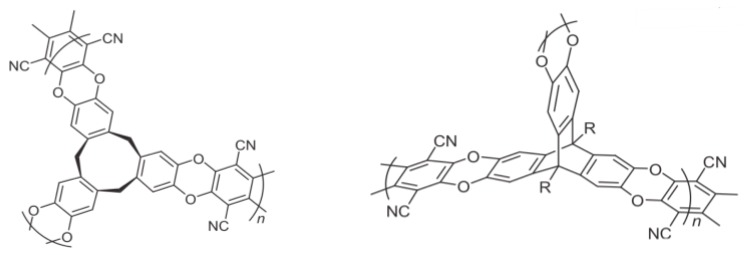
Basic structure of CTC-PIM, cyclotricatechylene-core polymer of intristic microporosity (**a**) and Trip(R)-PIM, a triptycene-core with variable R groups (**b**).

Using the same synthetic strategy, other types of PIMs were also synthesized, such as those composed of rigid and distorted macromolecules with fused-ring components, as shown in Figure 7. The Brunauer–Emmett–Teller (BET) surface (*S*_BET_) area, based on the BET model for N_2_ absorption, is 830 m^2^/g and the H_2_ adsorption ability at 77 K and 1 bar is ~1.43 wt % for cyclotricatechylene CTC-PIM polymers of Figure 7a [77]. Other PIMs based on same reaction mechanism with the similar monomers, and similar mild conditions for synthesis such as PIM-7, HATN-PIM, and Porph-PIM, were also studied by McKeown et al. [78,79]. These investigators found that, for similar structures, both *S*_BET_, and the H_2_ uptake ability are also similar. One PIM had a distinctly different structure made of non-planar units is Trip(R)-PIM (Figure 7b). This polymer results from a synthesis of triptycene subunits prepared through a dibenzodioxane formation reaction of hexahydroxyltriptycene and tetrafluoroterephthalonitrile [80]. In this system, the surface area is much higher, ~1760 m^2^/g, and the corresponding H_2_ uptake capacity is 1.79 wt % at 77 K and 1 bar. Recently, a set of three triptycene based microporous polymers (TMPs), created through Friedel–Crafts alkylations between tryptocene and multi-bromomethyl substituted benzenes were prepared. These systems share the rigid backbones of PIMS with flexible benzylic bonds. The best performing polymer, TMP-3, exceeded the performance of other PIMS, with a total surface area (*S*_BET_ = 1372 m^2^/g), significant micropore volume (0.163 mL/g), and 4.42 wt % H_2_ uptake at 77 K/1 bar [81]. The advantage of these three systems two PIMs is that all three show ultra microporosity with the range of 6–8 Å, which is needed to retain H_2_ molecules at room temperature.

**Figure 8:**
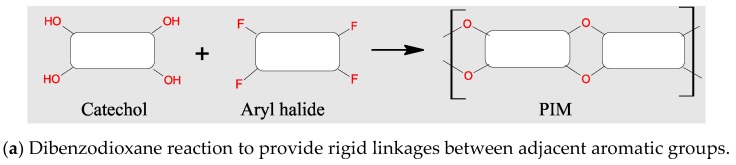

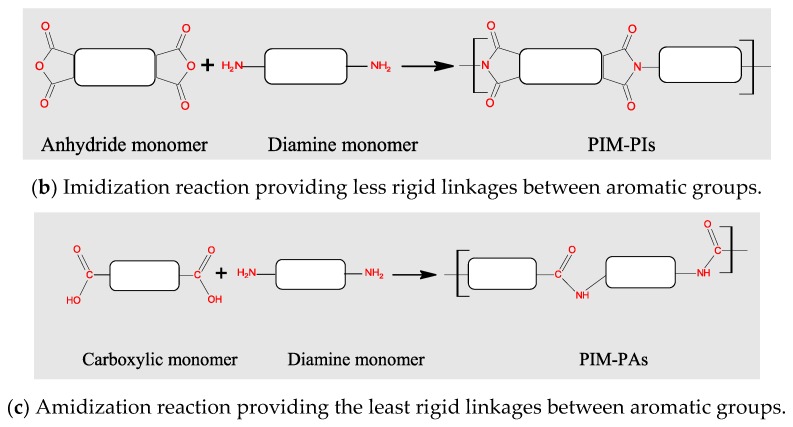
Schematic synthetic strategy for polymers of intrinsic microporosity (PIMs) produced through condensation under mild reaction conditions: K_2_CO_3_, DMF, 60–120 °C (Reproduced with permission from [84]. Copyright @ 2016 Elsevier).

In addition to PIMs synthesized via the dibenzodioxane reaction, other PIMs were made via imidization and amidization, using amine or amide precursors, as shown in schematic reaction diagrams in Figure 8 [82,83]. The synthesis of PIMs and its applications was systematically reviewed by Ramimoghadam et al. [84]. Generally speaking, polymer chains in PIMs must contain aromatic heterocyclic ladder components in the polymer backbone that restricts the free rotation of the backbone, preventing dense packing, and therefore retaining high porosity.

Different monomers are used to generate different PIM polymers. The complete list of monomers for all three types of reactions can be found in [84]. Here we give an overview of the PIM polymers produced by these three synthetic pathways, in terms of the surface area and hydrogen storage capacity. From the general structures of the products in Figure 8, it is seen the first PIM is most rigid due to the restriction of rotation by two –O– bonds in the backbone, while the third is least rigid due to the free rotation of –CO– and –NH– bonds. The corresponding internal surface area of the first type of PIM is 120–1760 m^2^/g, and that of PIM–PIs is 551–1407 m^2^/g and that of PIM–PAs is 50–156 m^2^/g, with surface area decreasing with increasing bond mobility. Therefore, the first type of synthesis is more interesting in terms of hydrogen storage and a variety of different monomers have been used to make PIM polymers of this type for H_2_ storage. Since hydrogen storage is more about meeting technical specifications, rather than methodology development, we hereby highlight the PIMs with highest surface area. The PIMs with the largest surface area are called star triptycene-based microporous polymer (STPs), which have structures similar to Trip(R)-PIM as in Figure 7b. STPs can reach as high surface areas as high as 2000 m^2^/g and H_2_ storage capacity at 77 K and 1 bar can reach 1.92 wt %. Therefore, PIMs with star-shaped structures possess higher specific surface area (SSA) and then higher H_2_ uptake, aided by their three-dimensional networks. As seem with HCPs, the best PIMs still fall significantly short of H_2_ adsorption achieved with the best MOFs at elevated pressures; however, PIMs outperform MOFs at 1 bar/77K, with H_2_ storage capacities up to 4.5 wt % vs. MOFs of > 2.5%. Both fall short of DOE targets.

### 3.3. Conjugated Microporous Polymers (CMPs)

**Figure 9:**
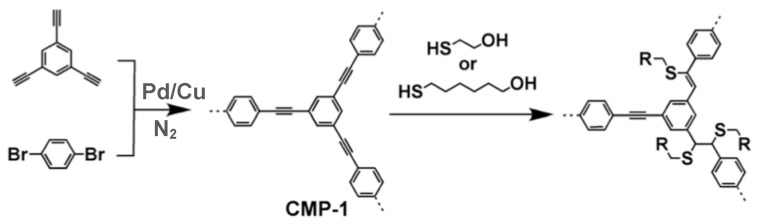
Basic synthetic route for conjugated microporous polymer-1 (CMP-1) and post-synthesis treatment. (Polymerization is promoted by Pd/Cu coupling under N_2_ atmosphere.)

Since the first discovery of conjugated microporous polymers (CMPs) by Cooper et al. [85,86], these polymers have been widely applied, including for H_2_ gas storage. The CMPs are amorphous polymers that are made of rigid blocks linked via π-conjugated bonds, and possess three-dimensional (3D) network structures, as shown in Figure 9. The diversity of building blocks and a wide availability of different reaction types, enable chemists to fine tune the microporosity in CMPs. The basic synthetic route for building 3D CMPs is the cross coupling of two different monomers, at least one of which has more than two reactive sites, as illustrated in Figure 9.

**Figure 10:**
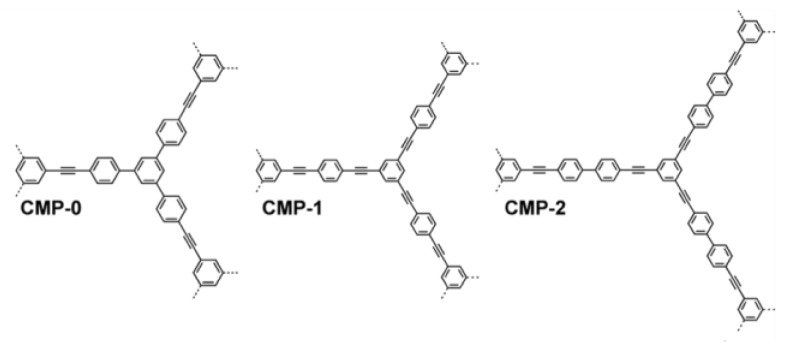
CMPs with varied length linkers between branch points.

The pore size can be varied by modifying the length of a linker between the molecules with three or more branching sites, as seen for the phenylethynylene-based CMPs in Figure 10. It is interesting to note that the longer the linker, the smaller the total surface area; e.g., CMP-0 has a surface area of ~1000 m^2^/g and CMP-1 and -2 have surface areas of ~ 34 and 634 m^2^/g, respectively.

Different phenyl monomers were used to synthesize CMPs with different specific surface areas (SSA) and pore size. For instance, Jiang et al. used 1,3,5-triethynylbenzene to make HCMP-1 with *S*_BET_ = 842 m^2^/g and pore size = 1.1–1.6 nm, and 1,4-diethynylbenzene to make HCMP-2 with *S*_BET_ = 827 m^2^/g and pore size = 0.9–1.6 nm [86]. Yuan et al. used the similar monomers, but a different metal catalyst, dicobalt carbonyl, to generate a serious of CMPs, POP-1, POP-2, POP-3, and POP-4, with smaller pore sizes (0.7–0.9 nm) but large surface areas (*S*_BET_ > 1000 m^2^/g) [87]. It is interesting to compare these series of CMPs in terms of H_2_ at 77 K as shown in Table 4. POP-3 has the largest *S*_BET_, so its H_2_ uptake is highest at this temperature even though the pore size is also largest, indicating that at low temperature it is surface area dominates the H_2_ adsorption ability.

On the other hand, POP-1 and POP-4 have the similar surface areas, but POP-1 has a higher H_2_ uptake due to the smaller pore size, even though the micropore volume is also smaller indicating the number of pores is about the same for the two systems. Therefore, when at 77 K, the specific surface area, rather than the pore size, contributes more to the H_2_ uptake. This is why most research groups have tried to make porous materials with as large surface area as possible. Unfortunately, there is no H_2_ adsorption data for this group of CMPs at room temperature and therefore, no comparison of H_2_ uptake for them under ambient conditions, when pore size would be expected to play a larger role.

Other polyaromatic-based CMPs, such as hexaphenylbenzene (HPB)-based porous organic polymers (HPOPs) [88], tetraphenylethylene (TPE)-based porous organic polymers (TPOPs) [89,90], phenolic-resin porous organic polymers (PPOPs) [73], and carbazole-spacer-carbazole type conjugated microporous polymers (P-1 and P-2) [91] have also been prepared and tested for H_2_ storage characteristics. The highest H_2_ uptake for HPOPs is HPOP-1 (*S*_BET_ = 1148 m^2^/g) with 1.50 wt % at 77 K/1.13 bar, for TPOPs (*S*_BET_ = 810 m^2^/g) is TPOP-5 with 1.07 wt % at 77 K/1.13 bar, for PPOPs is PPOP-3 (*S*_BET_ = 880 m^2^/g) with 1.28 wt % at 77 K/1 bar and for P-1/2 is P-2 (*S*_BET_ = 1222 m^2^/g) with 1.66 wt % at 77 k/1 bar. There is no significant improvement in terms of H_2_ adsorption for these different modified CMPs, and all fall far short of DOE specifications.

Recently, three novel CMPs were synthesized, based on a 4,4-difluoro-4-bora-3a, 4a-diaza-s-indacene (BODIPY) core coupled with 1,3,5-triethynylbenzene, named BDT1-3. The charged non-metal sites, in addition to high microporosity lead to improved H_2_ adsorption, particularly for BDT3, which had a total surface area of 101 m^2^/g, an average pore size of 0.7 nm, and 2.2 wt % uptake at 77K. While these values need still to be improved, presence of charged non-metals on the polymer backbone and small pore size of this system are promising [92].

In addition to varying the structure of the monomers, the microporosity can also be controlled by varying the percentages of building block precursors, as well as experimental conditions. For instance, conjugated carbazole backbone was spaced and linked by different length of alkenes to modify the pore structures. However, the BET surfaces were very similar (~800 m^2^/g) and the highest H_2_ uptake is 1.33 wt % at 1.0 bar/77 K [93]. Most pure organic CMPs possess internal surface of ~ 1000 m^2^/g or less, and the H_2_ uptake is around 1.0 wt % at ~77 K/1 bar [94,95,96]. In contrast, the Li doped CMP, with a relatively low surface area of 795 m^2^/g, has a much higher H_2_ uptake (~6.1 wt % at 77K/1 bar) than pure organic CMPs [97], due to the charge-induced dipole interaction between H_2_ and Li atoms as described in the Background Considerations section, and hence higher adsorption enthalpy. Unfortunately, Li ions tend to aggregate in the CMPs. As an improvement, another format of Li^+^, methyllthium (MeLi) doped in a naphthyl-containing conjugated microporous polymer (N-CMP) can bring excess H_2_ uptake to 6.5 wt % at 77 K/80 bar [98]. Halides, specifically Cl and Br have been incorporated into the amine-based CMPs, borazine-linked polymers (BLPs): BLP(Cl) and BLP(Br). BLP-2(Cl) to yield specific surface areas of up to 1174 m^2^/g and H_2_ uptake of 1.30 wt % at 77 K/1 bar, while BLP-2(Br) has specific surface area of 849 m^2^/g an H_2_ uptake of 0.98 wt % at the same conditions [99]. Other organic heteroatomic systems are tried as well. For instance, nitrogen-rich conjugated microporous polymers (N-CMPs) and nitrogen-rich azo-bridged porphyrin conjugated microporous networks (N-Azos) have been synthesized and yielded *S*_BET_ up to 485 and 675 m^2^/g with H_2_ uptake of 1.02 and 1.15 wt % respectively at 77K/1bar [100,101]. Thiophene-based conjugated microporous polymers (ThPOPs) have been synthesized and tested for H_2_ storage [102]. Among them, ThPOP-5 that retains both high BET surface (*S*_BET_ = 1300 m^2^/g) and micropores (*V*_micro_ = 0.28 cm^3^/g) can uptake H_2_ gas as high as 2.17 wt % at 77 K/1 bar. Incorporating organic groups such as azo and thiophene, increases the polarity of adsorption site, in addition to modifying the porosity. However as seen in Table 2, the adsorption enthalpy of the dipole-induced dipole (~0.6 kJ/mol) is much smaller than those of the charge-induced dipole (~6.8 kJ/mol) and the charge-induced quadropole (~3.5 kJ/mol). Therefore, it seems like that Li^+^ ions enhance H_2_ storage capacity more significantly than other main group elements investigated, with H_2_ storage capacity for Li^+^ ion systems exceeding the performance of the MOFs at 77 K/1 bar.

### 3.4. Porous Aromatic Frameworks (PAFs)

**Figure 11:**
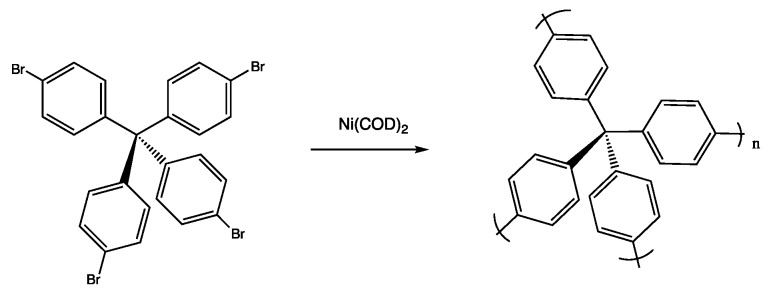
Basic synthetic route for porous aromatic framework-1 (PAF-1) (cross-coupling is promoted by Ni(0) catalyzed Yamamoto-type Ullman reaction under inert atmosphere.).

A major breakthrough in the use of highly porous organic polymers for H_2_ storage, was the discovery of a porous aromatic framework (PAF-1), which was synthesized via a cross coupling reaction of the tetrahedral building block tetrakis(4-bromophenyl)methane as shown in Figure 11.

The PAF-1 retains the highly porous networks that characterize MOFs and COFs, but with an amorphous structure, so it is classified as a polymer. PAF-1 not only has a very high internal surface area (~5600 m^2^/g), comparable to MOFs, but also is very thermally and hydrothermally stable, similar to COFs. Therefore, it combines the advantages of both MOFs and COFs. Due to its high specific surface area, the absolute and excess H_2_ uptake ability for PAF-1 can reach as high as 10.7 wt % and 7.0 wt %, respectively, at 77 K and 48 bar [103]. More recently, other larger tetrahedral molecules with different building blocks have been used to synthesize new PAFs, also prepared via Yamamoto homocoupling reactions [104,105]. For instance, Yuan et al. developed a series of PAFs, porous polymer networks (PPNs), using tetrahedral precursors, X(C_6_H_4_Br)_4_, where X = C, Ge, Si, etc. [105]. Among them, the PPN-4, which was developed from X = Si, has internal surface as high as 6461 m^2^/g, comparable to that of MOFs and COFs. The excess H_2_ adsorption can go as high as 9.1 wt % at 77 K and 55 bar. There is no H_2_ adsorption data reported at 298 K. However, H_2_ absorption is expected to drop to a low value at ambient temperature, due to the very weak van der Waals interaction between H_2_ molecules and hydrocarbon network. Another approach along this line is that organic linkers with different lengths were inserted between the tetrahedral building blockers to obtain different pore sizes. For example, by inserting diphenylacetylene (DPA), 1,4-diphenyl-buta- diyne (DPB), 1,4-bis (phenylethynyl) benzene (BPEB), or 1,4-bis (phe-nylbutadiynyl) benzene (BPBB) linkers, Wu et al. [106] synthesized a series of porous aromatic frameworks (PAF-322, PAF-324, PAF-332, and PAF-334). The size of the building unit in PAFs was significantly expanded and so were the pore size and internal surface area. The total H_2_ uptake can go as high as 63.96 wt % at 77 K/100 bar, and excess H_2_ uptake can reach 10.69 wt % at 77 K/20 bar. In particular, the total H_2_ uptake of PAF-334 at 298 K/100 bar is simulated to be 16.03 wt %. However, it is worthwhile to mention that the above results are the simulation results, not experimental results. Furthermore, due to the large open pores inside the polymer, the volumetric capacity of H_2_ storage for these PAFs is only approximate 9.0 g/L at 298 K/120bar, far below the DOE criteria (40 g/L). Similarly, theoretical simulations on designing up to 115 organic PAF-XXXs show that the weight storage capability of H_2_ can reach 5.9 wt % at 298 K/100 bar, while the volumetric capacity of H_2_ storage can only reach 7.9 g/L [107]. Therefore, simply increasing the pore size via different synthetic strategies might not be the right direction to make highly porous polymers that meet the DOE standards for H_2_ storage. 

In additional studies, several metals have been added to PAFs to enhance their H_2_ adsorption capacity. Similar to that in conjugated microporous polymers (CMPs), doping PAF-1 with 5% of Li (Li@PAF-1) significantly increases ultra micropore content (<10Å size) and possible adsorption enthalpy, and hence increases H_2_ adsorption ability by 22% [108]. Lithium ion doping in the PAF derivative, PAF-18-OH, also increases the polymer’s H_2_ adsorption [109]. In a related simulation, lithium-decorated fullerenes (Li_6_C_60_) were impregnated in PAFs and H_2_ uptake was predicted to be 5.5 wt % at 77 K/1 bar [110]. Addition of magnesium alkoxide was also studied by simulation, and is predicted to enhance the interaction between H_2_ and polymer pores, yielding a predicted H_2_ uptake of 7.12 wt % at 298 K and 100 bar [111]. Note that this is approaching the DOE 2020 targets for room temperature performance. Similar to what was observed for CMPs, a nitrogen-rich porous aromatic framework (N-PAF) was also developed with the *S*_BET_ = 1790 m^2^/g and H_2_ uptake up to 1.87 wt % at 77 K/1 bar [112]. Based on this study, the nitrogen substitution does not improve material performance.

Recently, Rochat et al. copolymerized the porous aromatic framework, PAF-1, with the polymer with intrinsic microporosity, PIM-1 [113]. By varying the relative contents of PAF-1 and PIM-1, they demonstrated that the PAF polymer retains a much larger internal surface area and hence a higher H_2_ store capacity. In fact, PAFs are the only polymer to date to show significantly higher H_2_ storage capacity than the most MOFs at high pressures. Data for performance at 1 bar is limited to a single study, showing H_2_ storage capacity similar to MOFs under these conditions.

### 3.5. Other Porous Polymers

Several emerging polymeric systems for H_2_ storage do not fit into the four classes above, including: (1) coordination polymers, (2) polypropylene gels, and (3) phosphorous-organic polymers. Coordination polymers can provide for both pores need for H_2_ storage and transition metals to enhance binding through d-orbitals. In a recent paper by Lyu et al. PCP-31 and -32 were created with chelated Cu^2+^ open metal sites incorporated into mesopores of 2.3–2.8 nm; despite the larger pore size, up to 10 wt % of H_2_ at 77 k/100 bar was adsorbed for the best performing system [114]. Polyphenylene gels are similar to other aromatic polymers, but lack non-aromatic linkers. A recent study demonstrated total surface areas of 219–674 m^2^/g for the polyphenylenes prepared, with assumed high microporosity. However, H_2_ adsorption data was not reported [115]. Ahmed et al. prepared three branched organic phosphate esters with azo linkages; despite low total surface values (up to 30.0 m^2^/g) and low total pore volume (up to 0.052 cm^3^/g), H_2_ adsorption at near ambient temperature (323 K/50 bar) is moderate (up to 0.66 wt %), signaling room for improvement with further structural modification [116].

### 3.6. Brief comparison of highly porous organic polymers with MOFs

**Figure 12:**
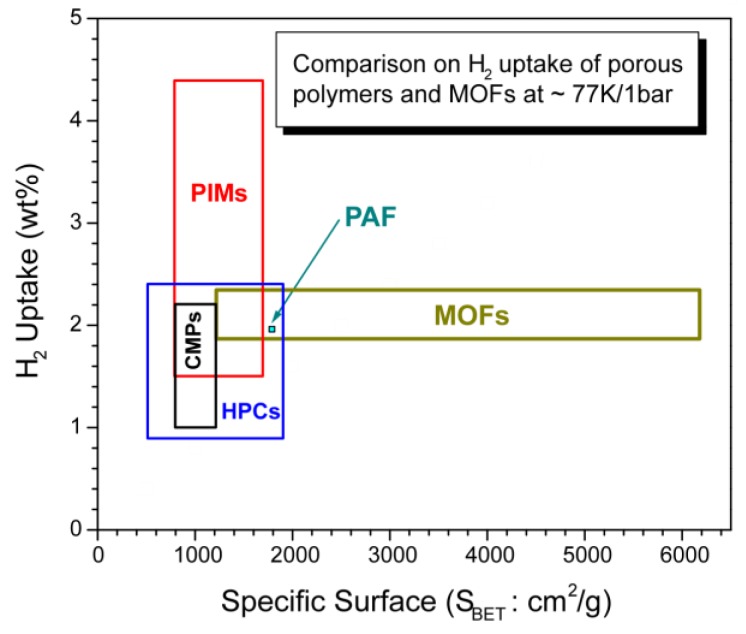
Brief comparison between highly porous polymers and MOFs in H_2_ adsorption.

To compare the performance of different materials in H_2_ storage, the experimental conditions for measuring the H_2_ uptake should be same, or at least similar, that is, under the same temperature and pressure. Unfortunately, the reported results available were obtained at different conditions. Although the temperatures for the experiments were at either 77 K or room temperature, the pressures for H_2_ measurement varied significantly, ranging from 1 bar to hundreds of bar. Such discrepancy in the experimental conditional makes the direct comparison impossible. Nevertheless, we selected the results under the similar conditions, close to 77 K/1bar, to make a rough comparison, as shown in Figure 12. Since the pressure is not exactly same, e.g. some 1 bar, and other 1.13 bar as seen in Table 3, we present the general ranges of pore size vs. H_2_ uptake, rather than individual definite data points in the figure.

It is interesting to see that polymers with intrinsic microporosity (PIMs) outperform the other highly porous polymers in H_2_ adsorption at ambient pressures. PIMS even outperform MOFs when under these conditions, despite the latter ones possess much larger internal surface and pore volumes. This analysis clearly indicates that the number of ultra micropores determines the total H_2_ adsorption, which is very consistent with the theoretical predictions.

## 4. Characterization of Porosity in Highly Porous Organic Polymers

As described above, ultra microporosity and adsorption enthalpy are key parameters for a highly porous organic polymer to adsorb large amounts of H_2_. The adsorption enthalpy can be measured by the adsorption dynamics. The porosity is traditionally characterized by the N_2_ gas adsorption method owing to the high relatively critical temperature of N_2_ gas, and well-defined theory. Normally, N_2_ gas is applied at different relative pressures from 10^–8^ to 1 to provide an adsorption isotherm, which describes the adsorption of N_2_ molecules over a wide range of porosity. To extract the information on porosity from H_2_ adsorption isotherms, different models have been proposed based on the mechanism of the micro-filling process in pores. The most widely used model is the Brunauer–Emmett–Teller (BET) model, which takes into consideration the multilayer adsorption of N_2_ molecules at liquid N_2_ temperature [117,118], yielding the total surface area. Alternatively, the Horvathe–Kawazoe (HK) method [119,120] and Dubinin–Radushkevich (DR) analyses [121,122] are often used to analyze micropore (*r* < 20 Å) volume and pore distribution at low pressure with the aid of the density functional theory (DFT) [123,124,125]. All three methods are based on the same experimental data: gas adsorption—normally, N_2_ gas adsorption at 77 K.

Unfortunately, N_2_ adsorption experiments have some intrinsic limitations when measuring ultra micropores (~Å) in highly porous polymers for H_2_ storage at ambient conditions. First, N_2_ adsorption methods do not effectively measure ultra micropores that are crucial to H_2_ storage due to large kinetic diameter of N_2_ molecules, 3.64 Å [126], which is comparable to the optimum pore sizes (~3.8 Å) for maximum H_2_ adsorption at room temperature. The adsorption of other gas media, such as CO_2_ and H_2_ has also been used to study the porosity [127]. However, no significant improvement in measuring micropores has been achieved, due to either the large size of CO_2_ molecules (3.3 Å), or the low critical temperature of H_2_ (32 K). Second, the N_2_ gas adsorption method provides isotherms at liquid N_2_ temperature (77 K), and is unable to generate the data needed to examine the H_2_ storage ability of a material at room temperature. Since pore volume in polymers significantly changes with temperature, the results obtained at 77 K might be very different from those at room temperature. In particular, ultra micropores (~Å) are much more sensitive to temperature, in that the expansion coefficient of those pores is almost ten times larger than the bulk value [128]. Therefore, some candidate materials ruled out by the N_2_ adsorption method at 77 K may be appropriate for H_2_ storage at room temperature, and vice versa. Finally, the adsorption of N_2_ at 77 K can cause a swelling effect or warp formation in the pore structure, giving hysteresis in the adsorption-desorption isotherm curve and therefore inaccurate information on the pristine pore sizes. 

**Figure 13:**
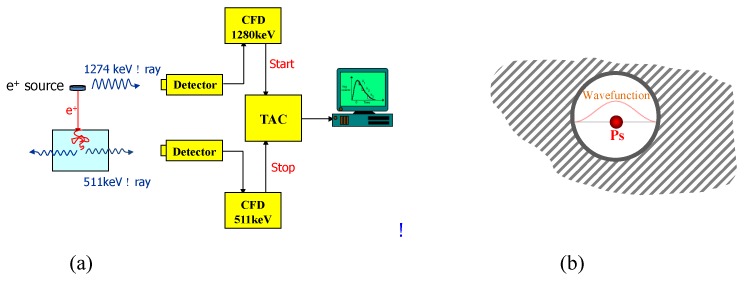
Schematic diagram of positron annihilation lifetime spectroscopy (PALS) technique: (**a**) setup and (**b**) mechanism.

Recently, another technique, positron annihilation lifetime spectroscopy (PALS) has been applied to study ultra micropores in H_2_ storage materials, providing a useful alternative to the N_2_ gas absorption method [84,108,129]. The positron is a particle with a positive charge and the same mass as an electron, and is generated from a positron source, normally a radioactive isotope ^22^Na. The schematic diagram for PALS technique and its mechanism is illustrated in Figure 13.

Figure 13a shows the general outline of the PALS setup. When a positron *e*^+^ emits from the radioactive source, an accompany ν ray with the energy of 1274 *keV* is given off simultaneously and detected by one detector, marking the birth of a positron. Then the positron will quickly slow down (at picosecond scale) and form a positronium (Ps). After diffusing and residing in a pore for a few nanoseconds, Ps will annihilate with the electron layer at the internal wall due to the overlap of Ps wave function with the above electron layer as shown in Figure 13b. The annihilation of Ps will give off another ν ray with the energy of 511 *keV* and detected by a second detector, marking the end of a positron. The time difference between these events is basically the lifetime of Ps, which is converted to the electronic signal via a time-amplitude-convertor (TAC) and recorded in a computer. The relationship between the lifetime of Ps and pore radius (r) is well described by the Tao–Eldrup equation as follows:

$$\tau =0.5ns{\left(1-\frac{R}{R+\Delta R}+sin\frac{2\pi R}{R+\Delta R}\right)}^{-1},$$

where τ is lifetime to Ps, R is the radius of a pore, and ΔR is an empirical value, 1.66Å, representing the thickness of electron layer on the internal wall of the pore [130]. 

The PALS technique is able to overcome the intrinsic limitations associated with the N_2_ adsorption method. First, Ps has same size as a hydrogen atom, with diameter of 1.06 Å, and is particularly sensitive to the pores with radii from 2–10 Å. This is the range of ultra micropores that theory predicts to be optimal in adsorbing H_2_ molecules at room temperature. Second, PALS measurements can be carried out at any temperature and hence can measure the porosity of highly porous polymers at room temperature. In addition, PALS can be performed in situ with H_2_ adsorption/desorption processes, thus providing dynamic information on H_2_ adsorption. Finally, Ps themselves will not create any swelling effect since both the positron and electron constituting Ps are leptons, and it is the quantum effect, rather than the classical space filling effect, that is used to obtain the pore size in PALS. 

Other techniques have also been used to study the porosity in porous polymers. For instance, ^129^Xe NMR spectroscopy, however, it was found Xe atoms exclusively occupy in large pores (>20 Å), not the micropores of interest [131]. Small angle X-ray scattering (SAXS) in combination with the N_2_ gas adsorption gives the internal surface area, however, this method still relies on the N_2_ adsorption isotherms with the limitations given above [83].

In summary, the technique best suited for characterizing ultra microporosity in highly porous organic polymers particularly for H_2_ gas storage is positron annihilation lifetime spectroscopy (PALS). However, PALS uses radioactive material, ^22^Na, which might limit its application even though its reactivity is relatively low ~30 μCi, and expertise with this method is not widespread.

## 5. Conclusions and Prospective Future

Porous organic polymers have their own advantages in H_2_ storage, for instance, the structures and porosity can be controlled by the use of different monomers and synthetic routes; the cost is relatively low and it is suitable for mass production, and normally, there is no heavy-metal incorporated, making the materials and processes friendly to the environment. These types of polymers store H_2_ molecules via physical adsorption through van der Waals interaction, and hence can release H_2_ as fuel under relatively mild conditions. Four types of highly porous organic polymers, hypercrosslinked polymers (HCPs), polymers with intrinsic microporosity (PIMs), conjugated microporous polymers (CMPs), and porous aromatic frameworks (PAFs) demonstrated high internal surface areas and good H_2_ uptake at cryogenic temperature. It is seen that the polymers originated from the star shaped precursors, such as Trip(R)-PIM and PAFs, can retain the highest specific surface area and outperform the other polymers generated from planar shaped precursors, and therefore, induce higher H_2_ uptake capacity under higher pressures. With the limited data published, only PIMs have been shown to have H_2_ storage capacities approaching 5 wt % at 77 K. However, due to the weak van der Waals interactions, the H_2_ adsorption enthalpy is only approximate ~6.0 kJ/mol for most carbon based polymers, and hence the H_2_ storage ability is very low under ambient temperature. Most of these materials would have an H_2_ absorption of ~1.0 wt % at room temperature, which is far below the targets specified by the DOE, despite of the fact that many highly porous polymers can store up to 10 wt % H_2_ at liquid N_2_ temperature (77 K) and high pressures (10 bar or more). 

To enhance the H_2_ adsorption ability at room temperature and ambient pressure, it is imperative to increase H_2_ adsorption enthalpy, in addition to high porosity. Theoretical considerations revealed two effective methods to achieve such goals: first, producing as many ultra micropores at several Å as possible; second, introducing some light metal ions, such as Li and Na ions, to create some charge-induced dipole interactions between H_2_ molecules and charge site. Ultra micropores, in contrast with macropores at nm scale or mesopores at μm scale, promote interaction between H_2_ molecules and pore walls, via the overlap of potentials from opposite walls and the quantum sieving effect that can be maintained up to 300 K, while larger pores can only have van der Waals forces between pore walls and H_2_. Unfortunately, most reports give only the content of overall porosity, which is probably composed primarily of macropores rather than ultra micropores, and therefore, the systems studied yield good to excellent adsorption result at 77 K, but do not perform well at room temperature. Nevertheless, there are some reports of highly porous polymers with high ultra micropore content. For instance, Zhang et al. [132] synthesized microporous polymer HTP-B using a hexaphenylbenzene-based triptycene monomer, in an attempt to introduce some ultra micropores under 10 Å. Although the exact ultra micropore content is not known, the H_2_ uptake is significantly higher than that of its counterpart, HTP-A that has no ultra-micropore content (1.09 wt % vs. 0.55 wt %). Therefore, future efforts should be directed to synthesizing highly porous polymers that have both high BET surface area and high content of ultra micropores. To further enhance the room temperature H_2_ absorption activity of these polymers, light metal ions can be doped into the materials. A third approach might be to add some transition metals to create so called spillover of H_2_ molecules. Spillover is a process by which H_2_ molecules dissociate and bond with C atom in *sp*^2^ hybrid orbital when catalyzed by some transitions metals, such as Pt^2+^ [133]. The spillover mechanism for H_2_ storage was initially hailed by many scientists since it was reported that the adsorption enthalpy of spillover could reach 10–30 kJ/mol, and the H_2_ adsorption could potentially exceed both 5.5 wt % and 50 g/L that were very close to the DOE criteria [134,135,136]. However, both theoretical and experimental study later confirmed that the spillover mechanism is not sufficient enough to generate onboard H_2_ storage [137]. Other concerns of spillover include the quick plague of the metal catalyst due to the oxidation reaction and irreversible hydrogenation [138] and the right position of the metal ions due to the amorphous structure of porous organic polymers. Therefore, it seems like spillover is not the right approach in this type of materials for H_2_ storage.

As a final note, the majority of the studies to date have focused on attaining the highest possible H_2_ capacities by using low temperature and/or high pressure conditions. These reviewers suggest that future studies would be better served in measuring H_2_ storage capacities at ambient temperature and pressure. This would not only make comparing data from different studies more uniform, it would also clearly illustrate progress toward achieving DOE H_2_ storage goals.

## Figures and Tables

**Table 1:** Major U.S. Department of Energy (DOE) Targets of Onboard Hydrogen Storage for Light Duty Vehicle.

	Year	2020	2025	Ultimate
Target				
Gravimetric capacity (wt %)		4.5%	5.5%	6.5%
Volumetric capacity (g/L)		30	40	50
Cost ($/kg H_2_)		333	300	266
Durability/Operability: Operating temperature (°C) Min/max delivery temperature (°C) Operational cycles Min/max delivery pressure (bar) Onboard efficiency		−40/60 −40/85 1500 5/12 90%	−40/60 −40/85 1500 5/12 90%	−40/60 −40/85 1500 5/12 90%
Charging/Discharging rate: System fill time (min) Min full flow rate ((g/s)/*k*W) Average flow rate ((g/s)/*k*W) Start time to full flow @ 20 °C (s) Start time to full flow @ −20 °C (s) Transient response at operating temperature 10%–90% and 90%–0% (based on full flow rate) (s)		3–5 0.02 0.004 5 15 0.75	3–5 0.02 0.004 5 15 0.75	3–5 0.02 0.004 5 15 0.75

**Table 2:** Dependence on distance and magnitude of different type of interactions between an H_2_ and adsorbent molecules (Modified from Ref. [35]).

Interaction type (Material – H_2_)	Energy dependence	Typical values (kJ/mol)
Charge – H_2_ quadropole	∝ 1/*r* ^3^	~ 3.5
Charge – induced H_2_ dipole	∝ 1/*r* ^4^	~ 6.8
Dipole – induced H_2_ dipole	∝ 1/*r* ^5^	~ 0.6
van der Waals	∝ 1/*r* ^6^	~ 5 – 6
Orbital interaction	<vdW radii	~ 20 – 160

**Table 3:** Hyper crosslinked polymers (HCPs) and their H_2_ uptake.

Monomer	Crosslinker	SSA (m^2^/g)	H_2_ Uptake	Ref.
VBC-DVB	VBC	1300–1900	3 wt % 77 K/15 bar, 1.5 wt % 77 K/1 bar	[52,53]
VCB	DVB	1500	1.59 wt % 77 K/1.13 bar	[65]
Carbazole bromophenyl- methanol	1,10-Phenantroline	1000	2.39 wt % 77 K/1 bar	[66]
Carbazoles	FDA	1000–1800	1.94 wt % 77 K/1 bar	[67]
Carbazoles	DCX	600–1900	0.9–17.7 wt % 77 K/1 bar	[59]
Polyanilines	CH_2_I_2_	~500	2.2 wt % 77 K/30 bar	[57]
Polypyrroles	CH_2_I_2_	~20–700	0.6–1.6 wt % 77 K/4 bar	[58]
Bismaleimides	DVB	841	0.82 wt % 77 K/1 bar	[68]
TPB	Cl	1783	1.91 wt % 77 K/1 bar	[69]
TPB	FDA	1059	1.58 wt % 77 K/1.13 bar	[70]
Benzene	FDA	1391	1.45 wt % 77 K/1.13 bar	[70]
Fluorene	BCMBP	1700	1.63 wt % 77 K/1 bar	[71]
TPE	FDA	1980	1.76 wt % 77 K/1 bar	[72]

VBC-DVB: vinylbenzyl chloride-divinylbenzene; VBC: vinylbenzyl chloride; DCX: dichloroxylene; DVB: divinylbenzene; FDA: formaldehyde dimethyl acetal; TPB: 1,3,5-triphenylbenzene; BCMBP: 4,4’-bis(chloromethyl)biphenyl; TPE: tetraphenylethylene.

**Table 4:** Comparison of specific surface area, pore size, and H_2_ uptake in porous organic polymers (POPs) (Adapted from [87])

CMPs	S_BET_ (m^2^/g)	Pore diamter (nm)	Micropore volume (cm^3^/g)	H_2_ uptake
POP-1	1031	0.77	0.378	2.78 wt % 77K/60bar
POP-2	1013	0.74	0.341	2.71 wt % 77K/60bar
POP-3	1246	0.88	0.448	3.07 wt % 77K/60bar
POP-4	1033	0.81	0.402	2.35 wt % 77K/60bar
